# Drought mitigation in cocoa (*Theobroma cacao* L.) through developing tolerant hybrids

**DOI:** 10.1186/s12870-021-03352-4

**Published:** 2021-12-15

**Authors:** Baby Juby, Janaki Seifudeen Minimol, Basura Suma, Adiyodi Venugopal Santhoshkumar, Joseph Jiji, Pottekkat Sidharthan Panchami

**Affiliations:** 1 Department of Plant Breeding and Genetics, College of Agriculture, Kerala Agricultural University, Thrissur, India; 2 Cocoa Research Centre, Kerala Agricultural University, Thrissur, India; 3 Department of Tree Physiology and Breeding, College of Forestry, Kerala Agricultural University, Thrissur, India

**Keywords:** Nitrate reductase, Proline, Superoxide dismutase, Logistic regression

## Abstract

**Background:**

Cocoa, being a shade loving crop cannot withstand long periods of water stress. Breeding for drought tolerance is the need of the hour due to change in climatic condition and extension of crop to non-traditional areas. Hybrids were produced by crossing four tolerant genotypes in all possible combination. The cross GV1 55 x M 13.12 didn’t yield any fruit due to cross incompatibility between these genotypes. Various biochemical parameters act as the true indicators to select tolerant and susceptible types. The major biochemical parameters considered after imposing stress included proline, nitrate reductase activity, superoxide dismutase content and glycine betaine.

**Results:**

The drought tolerant hybrids were having high amount of proline, superoxide dismutase enzyme and glycine betaine content. Normally, plants having drought stress show low amount of nitrate reductase activity. However, in case of hybrids, the drought tolerant hybrids were having higher NR activity than the susceptible hybrids. The highest amount of NR was found in the control plants kept at fully irrigated conditions.

**Conclusions:**

This experiment showed the role of different biochemical enzymes and osmolytes in giving tolerance to plants during drought stress. Logistic regression analysis selected proline and nitrate reductase as the two biochemical markers for identifying efficient drought tolerant genotypes in the future breeding programmes.

## Background

Cocoa is a high industrial valued crop that originated in humid tropics of rain forest and produce cocoa beans which is the only source for chocolate. Cocoa plays a significant role in economics of both producing and consuming countries. In the whole world cocoa is cultivated by around five million farmers and contributes to the subsistence of 40–50 million people [1]. The continuous climate change and limitations in availability of water in several regions of the world, creates a serious threat to plantation industry, especially cocoa as the crop is drought sensitive. Even though cocoa is cultivated under rainfed condition in many cocoa growing countries, inconsistency in rainfall pattern forms the major constrain. It is estimated that a minimum of 24 l of water/ 4–5 days interval is required for the plant to express its maximum potential [2]. The main reason behind this is that it has a very shallow tap root system which enables it to absorb water from surface layers only. The growth and yield of cocoa is influenced by a number of environmental factors, particularly rainfall, temperature and water stress [3].

Drought is defined as a decrease in water inputs or precipitation in an agro/ecosystem over time that is sufficient to result in soil water deficit [4]. Drought stress is the most common environmental stress causing threat to successful production of crop plants. There is a growing concern about the global increase in temperature and simultaneous increase in potential evapo- transpiration [5]. Plant water demand may result in increased drought stress during the day and a further deterioration of climatic condition for cocoa. Drought stress normally decreases plant growth by stressing various physiological and biochemical processes in plant, so these parameters can be used as an indicator of stress responses in plants. The plant response to drought stress are usually studied based on physiological parameters, but in recent studies biochemical and antioxidant responses also been proposed as appropriate indicators of drought stress in plants [6, 7].

Moreover, due to the increased rate of consumption of cocoa in chocolate industry, the cultivation is extending to non-traditional area. In such area acute water shortage is the most important problem to be addressed. Hence to cope up with the changing climatic scenario and cropping pattern it is essential to breed genotypes that can tolerate water stress. The screening of genotypes based on biochemical parameters helps in developing drought tolerant hybrids which can be used in further breeding programmes. The objective of the present study is to screen and identify cocoa genotypes which can tolerate drought and the role of biochemical parameters in drought stress so as to develop cocoa varieties suitable for drought prone areas.

## Materials and methods

### Experimental site

The hybridization work was conducted at Cocoa Research Centre (CRC), Kerala Agricultural University, India, using four genotypes identified as tolerant to drought in a preliminary study [8] as parents (Table 1). They were crossed in diallel out of which, the cross GV1 55 x M 13.12 yielded no fruits due to cross incompatibility. The pods matured approximately within 5–6 months; mature pods from each cross were harvested separately and raised in the nursery. Since parents are in heterozygous condition F1 population itself was segregating. Hence each individual was considered as separate hybrids.

**Table 1:** List of parents used for hybridisation

Sl No	Accession No.	Source
1	M13.12	Progeny of pods from Vittal
2	G1 5.9	T76/1224/1201 (Amazon)
3	G II 19.5	Progeny of pods from Nileshwar
4	G VI 55	Progeny of pods from Cadbury farm, Chundale

### Drought stress imposition and management

This study was carried out during 2019–20 in a research greenhouse at Cocoa Research Centre, Vellanikkara located in Thrissur (10° 32′ N, 76° 17′ E). Based on initial vigour, hybrids were selected and subjected to moisture stress following gravimetric method [9]. Seedlings of 5 month old with an average of 15 leaves were selected for drought screening in green house. Each progeny is replicated three times and used in the experiment. Initial trials in CRC indicated that cocoa cannot tolerate water stress less than 40% field capacity [10]. Hence, 40% field capacity was maintained for 2 weeks.

A control was also kept at fully irrigated condition representing each of the crosses. Based on the percentage of leaves retained after the drought imposition for 2 weeks, morphological classification of these hybrids was done based on the score chart (Table 2). The humidity and the temperature of the mist chamber were recorded using Berlin’s psychrometer on daily basis. Percent of leaves retained was calculated using the formula.

$$\mathrm{Percentage}\ \mathrm{of}\ \mathrm{leaves}\ \mathrm{retained}=\frac{\mathrm{Number}\ \mathrm{of}\ \mathrm{leaves}\ \mathrm{retained}}{\mathrm{Total}\ \mathrm{number}\ \mathrm{of}\ \mathrm{leaves}}\times 100$$

**Table 2:** Score chart depicting the leaves retained in the hybrids

Sl No.	Percentage of leaves retained	Classification
1	0–10	Highly Susceptible (HS)
2	10.1–40	Susceptible (S)
3	40.1–70	Tolerant (T)
4	More than 70	Highly tolerant (HT)

### Analysis of biochemical parameters

Biochemical analysis was carried out using standard procedures after 2 weeks of stress imposition. The parameters considered were proline (μg/g) [11], Nitrate reductase activity (mmol nitrate/g/hr) [12], superoxide dismutase (units/mg protein/g) [13] and glycine betaine (μmol/g) [14]. Analysis of variance was done for biochemical analysis for all selected hybrids following completely randomised design (CRD).

### Corelation and path coefficient analysis

The correlation coefficients were calculated to determine the degree of association of characters with percentage of leaves retained. The genotypic coefficients of correlation between character pairs were determined by using the variance and covariance components [15]. The direct and indirect effects of various biochemical parameters was estimated by path coefficient analysis by using the simple correlation coefficient [16, 17] and the direct and indirect effects were grouped into very high (> 1.00), high (0.30–0.99), medium (0.20–0.29), low (0.10–0.19) and negligible (0.09–0.00) [18].

### Binary logistic regression analysis

Binary logistic regression was carried out to find the relationship between the dependent biochemical parameters over the independent variable, the number of leaves retained. Improvement in selection over the base population was found by the equation,

$$\mathrm{Per}\ \mathrm{cent}\ \mathrm{improvement}\ \mathrm{over}\ \mathrm{base}\ \mathrm{population}=\frac{\mathrm{Exp}\left(\mathrm{B}\right)}{1+\mathrm{Exp}\ \left(\mathrm{B}\right)}\times 100$$

## Results

### Selection of hybrids and classification

A total of 1505 hybrid seedlings were raised in the nursery. Based on initial vigour 120 hybrids were selected representing 11 crosses. After imposition of water stress for 2 weeks the percentage of leaves withered was calculated and hybrids were classified as highly tolerant (HT), tolerant (T), susceptible (S) and highly susceptible (HS) as per the score chart given. Table 2. Different biochemical characters recorded from 120 hybrids are statistically analysed and depicted in Table 3.

**Table 3:** Biochemical parameters of cocoa hybrid seedlings

Sl. No.	Hybrids	Reaction to drought	Proline (μg/g)	NRA (mmol nitrate/g/hr)	SOD (units/mg protein/g)	Glycine betaine (μmol/g)
**(I). M13.12 X G I 5.9**						
1	H1	S	269.08	1.34	0.208	6.30
2	H2	T	695.36	5.60	0.373	7.48
3	H3	S	181.96	4.16	0.186	4.42
4	H4	S	219.44	2.28	0.205	6.03
5	H5	S	247.51	2.14	0.209	5.28
6	H6	S	298.52	3.82	0.210	6.22
7	H7	S	304.19	3.24	0.207	6.21
8	H8	S	354.34	4.04	0.188	6.15
9	H9	T	996.41	8.59	0.327	6.35
10	H10	T	440.93	11.92	0.381	6.77
11	H11	T	452.91	12.59	0.317	7.03
12	H12	T	539.50	6.94	0.351	7.09
13	H13	T	446.25	6.25	0.318	7.43
14	H14	S	293.06	4.03	0.210	5.33
15	H15	S	254.43	2.43	0.159	6.27
16	H16	S	310.02	2.19	0.128	5.25
17	H17	S	174.24	0.51	0.210	5.39
18	H18	S	167.18	2.56	0.209	6.04
19	H19	S	175.86	3.48	0.211	6.26
20	H20	T	418.28	7.12	0.318	6.78
21	H21	S	290.00	4.22	0.164	5.71
22	H22	S	189.29	3.35	0.212	5.94
23	H23	S	173.17	4.03	0.147	5.32
24	H24	T	468.86	5.85	0.351	7.99
25	H25	T	438.26	8.39	0.325	7.43
26	H26	T	504.07	5.08	0.321	11.64
27	H27	HT	1105.64	10.93	0.364	10.43
28	H28	T	479.56	7.13	0.343	7.81
29	H29	T	479.54	5.16	0.319	6.43
30	H30	S	340.35	1.02	0.191	5.36
31	H31	T	490.21	5.11	0.345	8.77
32	H32	S	209.81	2.65	0.164	5.32
	Control		61.33	17.03	0.023	3.22
	CV (%)		4.83	14.60	16.93	10.36
	CD	0.05%	30.54	1.12	0.07	1.12
**(II). M13.12 X G II 19.5**						
33	H33	T	671.38	6.36	0.316	9.46
34	H34	T	547.57	6.79	0.315	9.68
35	H35	S	359.00	2.70	0.195	6.73
36	H36	S	459.65	2.07	0.203	6.06
37	H37	T	523.52	11.74	0.319	8.79
38	H38	HT	2710.82	7.16	0.332	9.52
39	H39	T	522.28	6.59	0.322	8.66
40	H40	S	224.46	3.62	0.197	7.34
41	H41	S	265.09	4.30	0.184	6.02
42	H42	T	619.43	8.52	0.311	9.35
	Control		75.73	19.98	0.064	2.84
	CV (%)		2.59	15.25	13.65	9.34
	CD	0.05%	34.72	1.55	0.06	1.30
**(III). M13.12 X G VI 55**						
43	H43	HT	2817.39	9.75	0.337	8.90
44	H44	HS	85.52	3.39	0.145	6.70
45	H45	S	163.72	3.02	0.166	5.29
46	H46	T	643.40	6.40	0.362	9.66
47	H47	S	111.90	3.49	0.215	6.98
48	H48	T	494.21	10.89	0.334	9.87
49	H49	S	400.96	4.12	0.193	6.87
50	H50	S	247.77	1.88	0.215	7.06
51	H51	S	170.51	4.05	0.215	7.13
	Control		65.60	17.27	0.035	2.82
	CV (%)		2.65	13.85	18.88	11.74
	CD	0.05%	25.98	1.24	0.08	1.53
**(IV). G I 5.9 x M 13.12**						
52	H52	T	536.70	8.45	0.259	8.10
53	H53	S	292.40	2.96	0.167	6.43
54	H54	S	181.17	1.81	0.172	5.88
55	H55	S	386.31	3.92	0.156	5.36
56	H56	S	235.78	3.88	0.173	6.57
57	H57	T	498.21	4.25	0.222	7.97
	Control		85.70	12.95	0.027	3.16
	CV (%)		6.01	15.02	18.31	10.21
	CD	0.05%	38.00	1.13	0.06	1.22
**(V). G I 5.9 X G II 19.5**						
58	H58	S	218.46	3.91	0.218	6.83
59	H59	S	333.96	1.41	0.217	7.18
60	H60	S	326.36	3.62	0.162	7.34
61	H61	S	311.71	2.09	0.166	7.32
62	H62	T	568.81	8.39	0.248	8.93
63	H63	T	706.01	8.66	0.241	9.52
64	H64	T	679.37	9.48	0.243	8.61
65	H65	S	325.70	4.00	0.213	7.11
66	H66	S	177.26	1.42	0.222	7.17
67	H67	T	699.35	9.05	0.237	8.32
68	H68	T	684.66	8.56	0.276	9.38
69	H69	S	196.48	4.03	0.173	7.28
70	H70	S	167.84	3.32	0.204	6.45
	Control		138.00	15.78	0.050	3.34
	CV (%)		6.84	8.68	18.23	9.82
	CD (0.05)		47.62	0.76	0.07	1.29
**(VI). G I 5.9 X G VI 55**						
71	H71	HT	1749.05	9.94	0.256	9.56
72	H72	S	319.70	4.53	0.227	7.05
73	H73	S	395.63	3.65	0.234	7.07
74	H74	HT	1555.89	9.83	0.254	8.85
75	H75	T	450.25	10.26	0.272	9.38
76	H76	HT	1126.96	10.41	0.266	8.63
77	H77	S	169.54	4.50	0.191	6.34
78	H78	S	224.99	4.28	0.218	6.30
	Control		101.33	16.61	0.066	3.18
	CV (%)		4.54	6.58	12.65	9.90
	CD	0.05%	35.13	0.82	NS	1.35
**(VII). G II 19.5 x M 13.12**						
79	H79	T	853.88	7.18	0.225	8.87
80	H80	S	325.03	4.62	0.177	6.78
81	H81	T	498.21	14.27	0.209	8.91
82	H82	T	692.69	8.54	0.197	8.70
83	H83	T	454.91	7.48	0.224	8.66
84	H84	S	269.08	5.27	0.181	7.07
85	H85	HT	2293.88	7.72	0.227	8.43
86	H86	S	374.99	5.20	0.182	7.06
87	H87	S	363.66	4.24	0.170	7.51
88	H88	T	625.80	11.82	0.199	10.08
89	H89	S	295.73	3.98	0.183	7.31
90	H90	HT	1689.10	10.63	0.205	8.89
91	H91	S	139.87	2.80	0.184	7.55
92	H92	S	303.72	5.09	0.180	7.20
93	H93	S	338.75	4.03	0.133	6.93
94	H94	T	447.59	6.90	0.217	9.17
95	H95	S	208.47	4.02	0.162	6.84
96	H96	HT	2011.47	7.80	0.215	9.55
	Control		95.47	15.49	0.042	3.51
	CV (%)		4.52	7.95	19.43	10.15
	CD	0.05%	43.84	0.89	NS	1.36
**(VIII). G II 19.5 x G I 5.9**						
97	H97	HT	1490.62	15.45	0.265	9.46
98	H98	S	169.18	4.76	0.209	7.71
99	H99	S	363.66	4.80	0.213	7.20
100	H100	S	215.67	5.13	0.199	7.90
101	H101	HT	2726.81	9.80	0.270	9.70
102	H102	T	454.25	9.90	0.248	11.31
103	H103	T	500.87	8.09	0.247	13.79
104	H104	T	543.50	8.86	0.239	9.80
105	H105	S	171.93	5.92	0.207	8.02
106	H106	S	162.52	5.49	0.154	7.57
	Control		90.13	18.81	0.017	3.33
	CV (%)		4.70	8.73	18.63	9.74
	CD	0.05%	54.47	1.16	NS	1.53
**(IX). G II 19.5 x G VI 55**						
107	H107	HT	1984.83	12.98	0.163	9.34
108	H108	S	339.69	6.65	0.106	7.18
	Control		69.33	16.66	0.065	2.43
	CV (%)		2.75	4.97	18.99	5.58
	CD	0.05%	72.38	1.11	NS	1.04
**(X). G VI 55 x G I 5.9**						
109	H109	S	308.91	4.12	0.185	6.93
110	H110	S	151.99	3.57	0.136	7.66
111	H111	T	424.27	6.68	0.207	8.94
112	H112	T	411.35	6.38	0.221	10.61
113	H113	T	520.85	7.24	0.207	9.93
114	H114	S	128.55	4.63	0.149	7.42
	Control		74.67	14.76	0.025	3.03
	CV (%)		7.23	6.15	17.34	11.23
	CD	0.05%	41.74	0.59	0.06	1.17
**(XI). G VI 55 x G II 19.5**						
115	H115	T	520.85	17.06	0.209	6.52
116	H116	S	182.50	4.10	0.140	4.42
117	H117	T	743.18	11.35	0.202	6.43
118	H118	HT	1354.75	8.76	0.227	8.50
119	H119	T	412.95	7.56	0.163	9.23
120	H120	T	507.53	6.98	0.173	5.67
	Control		94.00	23.05	0.032	3.18
	CV (%)		5.21	10.22	18.49	8.92
	CD (0.05%)		57.48	1.74	NS	1.08

CD (critical difference is used to compare means of different treatments that have equal number of replications)

### Effect of water stress on proline content

In all the 11 crosses, all the tolerant and highly tolerant hybrids indicated high proline content as compared to the susceptible hybrids. The control recorded minimum amount of proline under full irrigated conditions (Table 3).

In the cross M 13.12 x G I 5.9, the highest content was found in hybrid H27 (1105.64 μg/g) and the lowest value was found in H18 (167.18 μg/g). The cross M 13.12 x G II 19.5 had ten hybrids out of which H38 showed the maximum content of proline of about 2710.82 μg/g. Lowest value was found in H40 (224.46 μg/g). In cross M 13.12 x G VI 55, the highest content was found in H43 (2817.39 μg/g) which was a tolerant hybrid and lowest in H44 of only about 85.52 μg/g of proline indicating its vulnerability to drought stress. The cross G I 5.9 x M 13.12 had six hybrids where H52 had highest proline content of 536.70 μg/g and H54 (181.17 μg/g) was having lower proline content. In the cross G I 5.9 x G II 19.5, out of 13 hybrids, H63 had the highest proline content of 706.01 μg/g and the lowest was found in H66 (177.26 μg/g). The cross G I 5.9 x G VI 55 had eight hybrids, and the highest content was found in H71 (1749.05 μg/g) while the susceptible hybrid H77 (169.54 μg/g) had the lowest amount. In cross G II 19.5 x M 13.12, the highly tolerant hybrid H85 (2293.88 μg/g) was having the highest amount while the lowest values were found in H91 (139.87 μg/g) which was a susceptible hybrid. In the cross G II 19.5 x G I 5.9, high values for proline was observed in H101 (2726.81 μg/g) and the lowest in H98 (169.18 μg/g) which was classified as susceptible. In the cross G II 19.5 x G VI 55), only two hybrids were there, one of which was highly tolerant and the other one was susceptible. The H107 was having the proline content of 1984.83 μg/g and the susceptible one, H108 was having 339.69 μg/g of proline. The cross G VI 55 x G I 5.9 was having six hybrids and H113 was having the highest value of proline, 520.85 μg/g and lowest value was found in H114 (128.55 μg/g). In the cross G VI 55 x G II 19.5, highest value was observed in H118 (1354.74 μg/g) while the lowest value was observed in H116 which was susceptible to drought having only 182.50 μg/g of proline.

When the progenies of all crosses were compared, it was seen that the content ranged from 85.52 μg/g in H44 (M 13.12 x G VI 55) to 2817.39 μg/g in H43 (M 13.12 x G VI 55).

### Effect of water stress on nitrate reductase activity (NRA)

Tolerant hybrids had high resistance to drought stress and were able to regulate the nitrate reduction activity even with less water. In this experiment, the cross G VI 55 x G II 19.5 recorded the maximum amount of enzyme activity of about 17.06 mmol nitrate/g/hr. in H115 which is a tolerant hybrid. However, the susceptible hybrids had very low content of NRA, the lowest being in H17 (0.51 mmol nitrate/g/hr) from the cross M13.12 x G I 5.9. The control plant on the other hand, had the highest value of NRA which was kept under 100% field capacity.

In the cross between M 13.12 x G I 5.9, 32 hybrids were obtained out of which the H11 showed the maximum nitrate reductase activity of about 12.59 mmol nitrate/g/hr. while the lowest value of nitrate reductase activity was observed in the susceptible hybrid H17.

The cross M 13.12 x G II 19.5 had hybrids having values as high as 11.74 mmol nitrate/g/hr. in H37 to as low as 2.07 mmol nitrate/g/hr. in H36.In cross M 13.12 x G VI 55, H48 was having the highest NRA content of about 10.89 mmol nitrate/g/hr. The lowest value was found in H50 with only 1.88 mmol nitrate/g/hr. In the cross between G I 5.9 x M 13.12, highest NRA value was found in H52 (8.45 mmol nitrate/g/hr) which was a tolerant hybrid while the lowest value was found in H54 (1.81 mmol nitrate/g/hr). The cross G I 5.9 x G II 19.5 had H64 with the highest NRA value of 9.48 mmol nitrate/g/hr. while the lowest value for NRA was 1.41 mmol nitrate/g/hr. found in H59.In the cross between G I 5.9 x G VI 55, the highest NR activity was found in H76 with 10.41 mmol nitrate/g/hr. which is a highly tolerant hybrid. The lowest NR activity was found in H73 having 3.65 mmol nitrate/g/hr. which is susceptible hybrid. In cross G II 19.5 x M 13.12, H81 showed the maximum NR activity of 14.27 mmol nitrate/g/hr. and the lowest value (3.98 mmol nitrate/g/hr) was observed inthe susceptible hybrid H89.The cross G II 19.5 x G I 5.9 had hybrid having the highest value in NR activity (H97) with a value of 15.45 mmol nitrate/g/hr. and the lowest (4.76 mmol nitrate/g/hr) was reported in H98.The cross G II 19.5 x G VI 55 had two hybrids, the high NR value was observed in the tolerant hybrid H107 (12.98 mmol nitrate/g/hr) and the low value of NR activity was observed in H108 (6.65 mmol nitrate/g/hr). In the cross G VI 55 x G I 5.9, the tolerant hybrids were having the high NR activity, the highest being H111 (6.68 mmol nitrate/g/hr), and lowest in H110 (3.57 mmol nitrate/g/hr). The cross G VI 55 x G II 19.5 had values as high as 17.06 mmol nitrate/g/hr. in H115 while the lowest value was observed in H116 having 4.10 mmol nitrate/g/hr. nitrate activity.

### Effect of water stress on superoxide dismutase

In cross M13.12 x G I 5.9, the highest value was observed in H10 (0.381 units/mg protein/g) while the lowest value was observed in H16 (0.128 units/mg protein/g). In the cross M 13.12 x G II 19.5, highest SOD value was found in H38 (0.332 units/mg protein/g) which is a highly tolerant hybrid while the lowest was H41 (0.184 units/mg protein/g). In the cross M 13.12 x G VI 55, the highest SOD value was found in H46 (0.362 units/mg protein/g). The lowest SOD value was in hybrid H45 (0.166 units/mg protein/g) which was highly susceptible. The cross G I 5.9 x M 13.12 had the highest SOD values of about 0.259 units/mg protein/g expressed in hybrid H52 while the lowest values was found in H55 (0.156 units/mg protein/g). In cross G I 5.9 x G II 19.5, the highest value was present in hybrid H68 (0.276 units/mg protein/g). The lowest value obtained was in hybrid H60 (0.162 units/mg protein/g). The cross G I 5.9 x G VI 55 had high SOD activity in hybrid H75 (0.272 units/mg protein/g) whereas lowest SOD value was found in H77 (0.191 units/mg protein/g). In cross G II 19.5 x M 13.12, SOD values was highest in H85 (0.227 units/mg protein/g). The lowest SOD value was found in H93 (0.133 units/mg protein/g). In the cross G II 19.5 x G I 5.9, hybrid H101 (0.270 units/mg protein/g) had the highest value and the lowest was found in H106 (0.154 units/mg protein/g). In the cross G II 19.5 x G VI 55, two hybrids were obtained in which the hybrid H107 was highly tolerant hybrid having high SOD content of 0.163 units/mg protein/g whereas the susceptible hybrid was having 0.106 units/mg protein/g of SOD activity. In the cross G VI 55 x G I 5.9, high SOD value was found in hybridH112 (0.221 units/mg protein/g). The low SOD value was found in hybrid H110 (0.136 units/mg protein/g). In cross G VI 55 x G II 19.5, the highest value was found in hybrid H118 (0.227 units/mg protein/g) while the lowest value was found in hybrid H116 (0.140 units/mg protein/g) which is a susceptible hybrid.

H10 (M13.12 x G I 5.9) recorded the high SOD content of about 0.381 units/mg protein/g which was a tolerant hybrid and the lowest content was observed in a susceptible hybrid, H108 (G II 19.5 x G VI 55) of about 0.106 units/mg protein/g.

### Effect of water stress on glycine betaine (GB)

When the hybrids of present study were analysed, the tolerant hybrid, H103 (G II 19.5 x G I 5.9) was having high amount of glycine betaine of about 13.79 μmol/g and the susceptible hybrid, H3 (4.42 μmol/g) of the cross M13.12 x G I 5.9 recorded the least. All the tolerant hybrids had high glycine betaine content as compared to the susceptible hybrids. However, the control which was kept under fully irrigated condition had least amount of glycine betaine among the crosses indicating the accumulation of glycine betaine under drought stress conditions. In cross M 13.12 x G I 5.9, the highest glycine betaine value was found in H26 (11.64 μmol/g) and the lowest value was observed in H3 (4.42 μmol/g). In cross M 13.12 x G II 19.5, the highest value was found in hybrid H34 (9.68 μmol/g) whereas the lowest value was observed in H35 (6.73 μmol/g). In cross M13.12 x G VI 55 the highest value was observed in H48 (9.87 μmol/g) and the H45 (5.29 μmol/g) recorded the lowest glycine betaine value.

In cross G I 5.9 x M 13.12, high GB values were observed in H52 (8.10 μmol/g) while H55 (5.36 μmol/g) had the lowest value. In cross G I 5.9 x G II 19.5, the highest value was observed in H63 (9.52 μmol/g) to as low as 6.45 μmol/g in H70.In cross G I 5.9 x G VI 55, the highest value was observed in hybrid H71 (9.56 μmol/g). Lowest values were observed in H73 (7.07 μmol/g). In cross G II 19.5 x M 13.12, highest GB value was observed in H88 (10.08 μmol/g) while the lowest value was observed in H80 (6.78 μmol/g). In the cross between G II 19.5 x G I 5.9, H103 (13.79 μmol/g) recorded the highest value while the lowest value was observed in H99 (7.20 μmol/g). The cross G II 19.5 x G VI 55 had two hybrids, the hybrid H107 had high GB value of 9.34 μmol/g and the other hybrid, H108 which is the susceptible one, had 7.18 μmol/g of GB. In cross G VI 55 x G I 5.9, H112 was having the highest GB content of about 10.61 μmol/g while the lowest value was observed in H109 (6.93 μmol/g). In the cross G VI 55 x G II 19.5, the highest value was observed in hybrid H119 (9.23 μmol/g) while the lowest value was observed in H116 (4.42 μmol/g).

### Corelation and path coefficient analysis

All the biochemical parameters estimated showed positive corelation with dependant variable (percentage of leaves retained). Among the biochemical characters, proline showed maximum correlation with the dependent variable, percentage of leaves retained (0.777) (Table 4).

**Table 4:** Correlation among drought tolerant contributing characters of hybrids

	V1	V2	V3	V4
**V1**	1			
**V2**	0.534^**^	1		
**V3**	0.353^**^	0.440^**^	1	
**V4**	0.458^**^	0.581^**^	0.441^**^	1

^**^Correlation significant at 0.01 level

**V 1 -** Proline (μg/g)

**V 2 –** Nitrate reductase activity (mmol nitrate /g/hr)

**V 3 –** Superoxide dismutase (units/mg protein/g)

**V 4 –** Glycine betaine (μ mol/g)

**Fig. 1:**
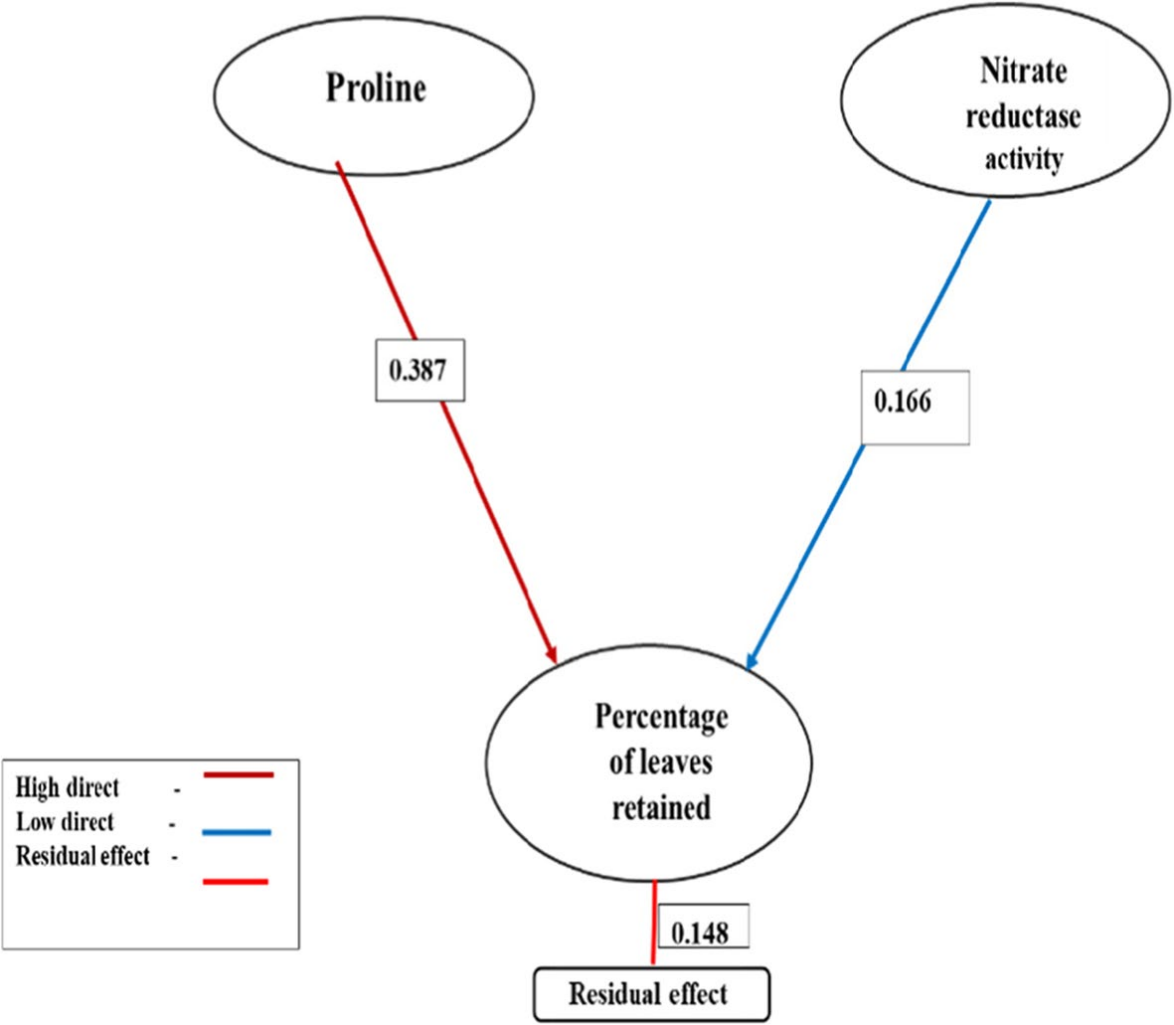
Path diagram for the biochemical characters having direct effects on percent of leaves retained

High positive effect on the percentage of leaves retained in path analysis result is a direct measure of plant’s tolerance to drought. It was expressed by proline (0.386) and low direct effect was shown by nitrate reductase activity (0.166) (Fig. 1). Negligible direct effects were found in superoxide dismutase (0.063), and negative but negligible effects were shown by glycine betaine (−0.016).

### Binary logistic regression analysis

The positive and comparable value of odds ratio Exp (B) and positive correlation indicated that proline content and NRA content had a positive correlation with drought tolerance and also these characters expressed a significant value of less than 0.05 which is the constant indicating the 95% accuracy with the results.

Based on the Exp (B) value from regression model, expressed percentage for drought tolerance over the base population was calculated and it was found that if selection is based on proline, a new population from the base population will express 51.55% improvement regarding the tolerance. In case of NRA, a new population will show 87.48% improvementover the base population (Table 5).

**Table 5:** Logistic estimate of characters influencing drought tolerance in cocoa

Variables	Coefficient	Standard error	Wald	Significance	Exp(B)	Expected per cent improvement over population (%)
**Proline**^a^	0.062	0.011	31.019	0.001	1.064	51.55
**Nitrate reductase activity**^a^	1.944	0.233	69.914	0.001	6.990	87.48

^a^Significant value less than 0.05

## Discussion

Drought stress is one of the important problems which adversely affects the performance of different crop plants worldwide. Various strategies are being adopted to cop up with adverse conditions. Development of drought tolerant cultivars is prerequisite to encounter the existing drought stress on sustainable basis. In the present study selection of hybrids were done based on initial vigor and stress imposition was carried out. In cocoa initial vigour is directly correlated to yield [19].

In general, plants employ various methods to promote tolerance to drought stress. To assess the degree of tolerance in plants, various drought-related indicators are used, such as morphological, physiological and biochemical parameters [20]. In the present study evaluation and screening of genotypes under drought stress were done based on biochemical parameters.

Proline is an important amino acid found in proteins. It has a significant contribution in drought tolerance. Under drought stress, a higher accumulation of proline was recorded in the study, which might be due to osmotic adjustment. Even within a single cross, highly tolerant as well as susceptible hybrids were found. This is because parents are heterozygous in nature [21]. The analysis of proline clearly indicated that resistant plants were high in proline content compared to susceptible ones. A clear-cut difference was observed between highly tolerant and susceptible genotypes indicating that proline plays an important role in drought tolerance of cocoa. High levels of proline enabled the plant to maintain low water potentials. Apart from acting as an osmolyte for osmotic adjustment, proline contributes to stabilizing sub-cellular structures (eg., membranes and proteins), scavenging free radicals and buffering cellular redox potential under stress conditions [22]. Similar studies were carried out in other crops. Under drought stress, there was a progressive increase in free proline in cotton plants [23] and a notable proline accumulation was observed in sugar beet leaves when drought stress became severe [24]. It has been already proved that proline is having direct correlation with drought stress [25, 26] as indicated in the present study. Proline in general was known to correlate with stress tolerance and has a direct effect on tolerance capability of plant [22] supporting our present findings.

Another important biochemical parameter contributes to drought stress is nitrate reductase activity. Water deficit induces an abrupt reduction in the uptake and nitrate flux rates from roots to leaves, preventing the mechanisms of NR protein synthesis induction and NR activity. The NR activity decline during water stress is mainly attributed to low NO3 - absorption and availability resulting from water uptake deprivation [27]. The present results indicated that the tolerant hybrids had high resistance to drought stress and were able to regulate the nitrate reduction activity even with less water. Usually, drought stress reduces the enzyme activity and that is the reason the amount of reductase enzyme was low in hybrids whereas in the control, it was more. NRase is closely associated with plant growth and development [28]. It is generally accepted that drought stress has a negative impact on plant’s photosynthetic activity, N concentrations, free amino acids or soluble protein contents accompanied with a decline of nitrate reductase activity in many plant species, such as maize [29], potato [30] and winter wheat [31]. The plants subjected to water stress produces less amount of total protein which results in a decrease in the synthesis of nitrate reductase activity caused by low nitrate flux [32]. The amount of NR enzyme generally decreases during drought stress and hence, the hybrids having more NR enzyme were more tolerant to drought stress and were able to regulate the nitrogen assimilation in plants [33]. Therefore, in the present study NR was found to be directly related to the dependent variable.

Reactive Oxygen Species (ROS) accumulation during stress greatly depends on the balance between ROS production and ROS scavenging mechanism [34]. When plants are subjected to any kind of stress, the cells have an increased production of reactive oxygen species (ROS) which in normal cases, is removed from time to time. Under stress, these become high in number and results in oxidative damage. These are removed by anti-oxidant systems which form the first line of defence which is superoxide dismutase. ROS-scavenging mechanisms were shown to have an important role in protecting plants against osmotic stresses [35].

In the experiment when plants were subjected to analysis, the tolerant and highly tolerant hybrids showed more amount of superoxide dismutase enzyme as compared to the susceptible hybrids and the control which was kept under fully irrigated condition had the least amount of SOD in it. This indicated that SOD will get accumulated under drought stress conditions and forms a defence system against the stress [36]. Superoxide dismutase (SOD) are the enzymes that forms the first line defense and catalyses the dismutation of O^2-^ radicals to H_2_ O and O_2_. Hence, the amount ofSOD increases with increase in stress conditions indicating it’sdirect relation to drought stress [37, 38]. Similar studies on development of water stress in *Curtilobumsolanum* and *Solanumtuberosum* due to over production of SOD in chloroplasts were also reported [39] which supported our findings.

Many plants accumulate compounds, termed compatible solutes, to cope with stress conditions. One of the most extensively studied compatible solutes is glycine betaine [40] Not only GB acts as an osmoregulator, but also stabilizes the structures and activities of enzymes and protein complexes, and maintains the integrity of membranes against the damaging effects of stress [41]. The role of glycine betaine to drought tolerance has been reported in many cases [42, 43]. Genes associated with glycine betaine synthesis in higher plants and microbes have been transferred into plants which do not accumulate glycine betaine, such as *Arabidopsis thaliana* [44], *Brassica napus* [45] *Persimmon* [46] and rice [47]. The metabolic engineering of glycine betaine biosynthesis in these plants improved the tolerance of transgenic plants to salt, drought and extreme temperature stresses [48]. When the levels of glycine betaine was correlated with the extent of increased tolerance, the accumulation of glycine betaine was found to be induced under stress conditions [49, 50].

**Fig. 2:**
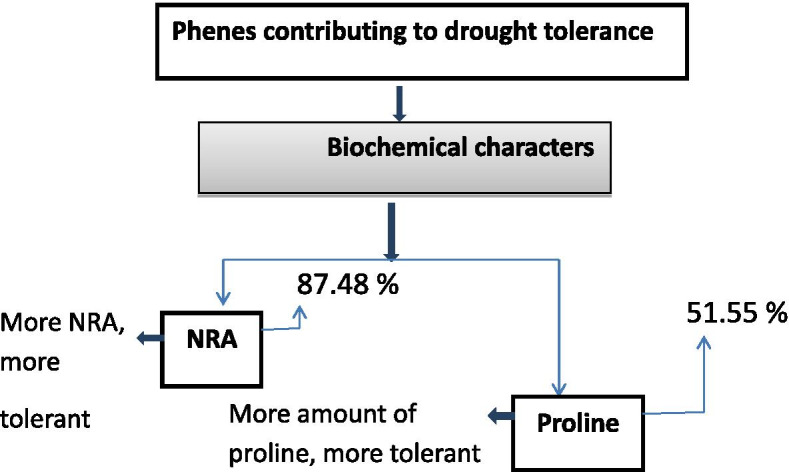
Biochemical phenes and their association with drought tolerance in cocoa

Hence, these biochemical parameters were reliable source to detect drought stress in cocoa. However, the correlation and path analysis revealed that proline and the nitrate reductase are the two important parameter that contribute to drought tolerance. To find out the actual relationship between the dependent variables (the biochemical parameters) and the independent variable (the number of leaves retained, a binary regression analysis was carried out. The results of present study indicated that the proline and nitrate reductase can be used as biochemical indicators in cocoa drought breeding programme (Fig. 2).

## Conclusion

The parameters proline and glycine betaine represented the osmolyte group whereas nitrate reductase and superoxide dismutase represented the enzyme group. In all the crosses, the content of proline was high in highly tolerant and tolerant hybrids as compared to susceptible hybrids. The control which was fully irrigated condition was having the least amount of proline. The glycine betaine also followed the same trend, as tolerant and highly tolerant hybrids were having more amount of glycine betaine as compared to susceptible hybrids and the control indicating that these two osmolytes accumulated only during water stress. In case of superoxide dismutase, the highly tolerant and tolerant hybrids were having high amount of superoxide dismutase as compared to the susceptible hybrids whereas the control was having the least amount of superoxide dismutase enzyme. In case of nitrate reductase activity, the highly tolerant and tolerant hybrids were having high amount of enzyme as compared to the susceptible hybrids. The control kept at fully irrigated condition was having the highest amount of nitrate reductase enzyme. The hybrids having high amount of nitrate reductase were more tolerant because generally, this enzyme reduces under drought stress. Based on corelation, path analysis and binary logistic regression analysis, proline and nitrate reductase can be used as reliable parameters in drought breeding programmes in the future.

## Data Availability

All data generated or analysed during this study are included in this article and its supplementary files.

## Abbreviations

NR: Nitrate reductase
NRA: Nitrate reductase activity
GB: Glycine betaine
SOD: Super oxide dismutase
HT: Highly tolerant
T: Tolerant
S: Susceptible
HS: Highly susceptible
ROS: Reactive oxygen species
CRC: Cocoa Research Centre
CRD: Completely randomised design
